# Opioid agonist therapy uptake among people who inject drugs: the findings of two consecutive bio-behavioral surveillance surveys in Iran

**DOI:** 10.1186/s12954-020-00392-1

**Published:** 2020-07-22

**Authors:** Mehran Nakhaeizadeh, Zahra Abdolahinia, Hamid Sharifi, Ali Mirzazadeh, Ali Akbar Haghdoost, Mostafa Shokoohi, Stefan Baral, Mohammad Karamouzian, Armita Shahesmaeili

**Affiliations:** 1 HIV/STI Surveillance Research Center, and WHO Collaborating Center for HIV Surveillance, Institute for Futures Studies in Health, Kerman University of Medical Sciences, Kerman, Iran, Haftbagh Highway, Kerman, 7616913555 Iran; 2 Department of Biostatistics and Epidemiology, School of Public Health, Kerman University of Medical Sciences, Kerman, 7616913555 Iran; 3 Modeling in Health Research Center, Institute for Futures Studies in Health, Kerman University of Medical Sciences, Kerman, 7616913555 Iran; 4 Department of Epidemiology and Biostatistics, University of California San Francisco, San Francisco, CA USA; 5 Department of Epidemiology & Biostatistics, Schulich School of Medicine & Dentistry, The University of Western Ontario, London, Canada; 6 Division of Social and Behavioural Health Sciences, Dalla Lana School of Public Health, University of Toronto, Toronto, Canada; 7 Department of Epidemiology, Johns Hopkins Bloomberg School of Public Health, Baltimore, MD USA; 8 School of Population and Public Health, Faculty of Medicine, University of British Columbia, Vancouver, BC Canada

**Keywords:** Opioid agonist therapy, People who inject drugs, Harm reduction, Surveillance, Iran

## Abstract

**Background:**

Opioid agonist therapy (OAT) uptake has been associated with multiple positive health outcomes among people who inject drugs (PWID). This study evaluated the pattern of OAT uptake among PWID in two consecutive national bio-behavioral surveillance surveys (2010 and 2014) in Iran.

**Methods:**

Data were obtained from two national bio-behavioral surveillance surveys (*N*_2010_ = 1783 and *N*_2014_ = 2166) implemented using convenience sampling at the harm reduction facilities and street venues in 10 geographically diverse urban centers across Iran. Multivariable logistic regression models were built to determine the correlates of OAT uptake for the 2014 survey, and adjusted odds ratios (AORs) along with 95% confidence intervals (CI) were reported.

**Results:**

The prevalence of OAT uptake decreased from 49.2% in 2010 to 45.8% in 2014 (*P* value = 0.033). OAT uptake varied across the studied cities ranging from 0.0 to 69.3% in the 2010 survey and 3.2 to 75.5% in the 2014 survey. Ever being married (AOR = 1.40; 95% CI 1.12, 1.75), having a history of incarceration (AOR = 1.56; 95% CI 1.16, 2.09), and human immunodeficiency virus (HIV) sero-positivity (AOR = 1.63; 95% CI 1.08, 2.50) were associated with OAT uptake. Conversely, PWID who reported using only non-opioid drugs (AOR = 0.43; 95% CI 0.26, 0.71) and those who reported concurrent use of opioid and non-opioid drugs (AOR = 0.66; 95% CI 0.51, 0.86) were less likely to uptake OAT.

**Conclusions:**

Although OAT uptake among PWID in Iran is above the 40% threshold defined by the World Health Organization, there remain significant disparities across urban settings in Iran. Importantly, the OAT services appear to be serving high-risk PWID including those living with HIV and those with a history of incarceration. Evaluating service integration including mental health, HIV and hepatitis C virus care, and other harm reduction services may support the optimization of health outcomes associated with OAT across Iran.

## Introduction

One of the most populated countries in the Middle East and North Africa (MENA) region is Iran where over one million people are estimated to use illicit drugs [1]. Moreover, the number of people who inject drugs (PWID) is estimated to be 280 per 100,000 population, about half of whom are infected with hepatitis C virus (HCV) and around 13.8% are living with human immunodeficiency virus (HIV) [2–4]. To reduce HIV, HCV, and other blood borne infections among PWID, a comprehensive and innovative harm reduction (HR) program has been implemented in Iran. Currently, healthcare facilities including voluntary counseling and testing (VCT) centers, HR centers for vulnerable women, shelters, prisons, antenatal clinics, and drop-in centers (DICs) provide onsite or outreach HR services to PWID. These services include but are not limited to opioid agonist therapy (OAT) by methadone, buprenorphine, or opium tincture, as well as needle and syringe programs (NSPs), VCT, and free condom distribution [5, 6].

Although buprenorphine maintenance therapy (BMT) and opium tincture are available in Iran, methadone maintenance treatment (MMT) programs are more common [1]. MMT programs were initially implemented in pilot projects in 2002; however, they were significantly scaled up in public and private clinical settings from 2003-2007. By September 2014, MMT was offered to PWID at 5744 private centers and 239 public centers supervised by State Welfare Organization, medical sciences universities, or prisons’ organization [7]. As of 2018, over 700,000 participants have received MMT programs in these centers [6]. The cost of MMT services is considerably lower in public centers [8].

OAT in PWID has been associated with several beneficial public health outcomes including decreasing the rate of fatal and non-fatal overdose, reducing the rate of HIV and HCV transmission, lowering the rate of violence, diminishing social costs associated with drug use, increasing PWID’s quality of life, and improving their employment status [9–13]. For PWID who are less connected to healthcare services, OAT could also represent a gateway to other services such as primary health care, HIV testing and counseling, antiretroviral therapy, and tuberculosis, HCV, and sexually transmitted infections (STI) care [14]. Our understanding of the prevalence and patterns of OAT uptake among PWID in Iran is limited. To monitor the impact of OAT programs in prevention of HIV, HCV, and hepatitis B virus (HBV), it is crucial to know the current level of OAT uptake among PWID in Iran. In response, we aimed to identify the prevalence and trend of OAT among PWID and determine the factors associated with OAT uptake using the data collected in two national consecutive bio-behavioral surveillance surveys conducted in urban settings across Iran in 2010 and 2014.

## Methods

### Study design and participant

Data from the 2010 (*N* = 1783) and 2014 (*N* = 2166) HIV national bio-behavioral surveillance surveys (BBSS) were used to assess the prevalence of OAT uptake among PWID in Iran. As PWID bear the highest burden of HIV on Iran, nation-wide surveys are conducted every few years to help monitor the trend of HIV and its related risk behaviors among this population and inform the national HIV response. The 2010 and 2014 surveys were conducted in 10 geographically diverse cities. Study participants were recruited from shelters, DICs, VCT centers, and street-based venues through outreach efforts. Eligible participants were 18 years or more and self-reported injection drug user for at least once during the previous 12 months. The details of the surveys are previously described [4, 15].

### Data collection

Data were collected through face-to-face interviews using a structured questionnaire consisted of sociodemographic characteristics, illicit drug use practices, sexual behaviors, knowledge about STI and HIV, history of incarceration, history of HIV testing, drug use treatment, and care-seeking behaviors. Individuals were given a monetary incentive equivalent to 5 USD for their participation.

### Dependent variable: OAT uptake

The outcome variable in the present study was OAT uptake. Participants were asked “Have you received any type of prescribed OAT including MMT, buprenorphine maintenance treatment, or treatment with opium tincture within the last month?” Responses were recorded as yes (coded as 1) or no (coded as 0).

### Covariates

These covariates included age at interview (≤35 or >35 years), gender (male or female), marital status (never married or ever married), monthly income levels (<200 USD or ≥200 USD), education levels (high school and above or less than high school), history of incarceration (yes or no), source of recruitment in the study (outreach or facility-based), substance type used in the past month (only opioid, only non-opioids, or opioids and non-opioids), self-perceived risk of HIV (yes or no), and HIV status (negative or positive). Opioids included opium, opium sap, heroin, crack, norchizak (i.e., a combination of several opioids with corticosteroids or benzodiazepines), tamchizak (i.e., a combination of industrial morphine hydrochloride and other synthetic drugs), and non-prescribed use of methadone, buprenorphine, and opium tincture. Non-opioids included hashish/grass/cannabis, marijuana, ecstasy, cocaine, and methamphetamine/crystal/shisheh.

### Ethics

The study protocol was reviewed and approved by the Research Review Board of the Kerman University of Medical Sciences (Ethics code No: IR.KMU.REC.597 and K/93/208), and Iran’s Ministry of Health.

### Statistical analysis

We first reported the prevalence of OAT uptake among PWID in two rounds. OAT uptake was also reported by subgroups of the covariates. Moreover, OAT uptake prevalence in two rounds was compared using two-sample proportion tests. Participants with missing responses to OAT uptake were excluded from the relevant estimates. Bivariable and multivariable logistic regression models for the survey were constructed to assess the correlates of OAT uptake among PWID based on 2014 data. Variables with a *P* value < 0.2 from the bivariable models were entered into the multivariable model. The final model was selected using a backward selection approach. As participants were recruited from different cities, each one was considered as a cluster and their clustering effects were adjusted using Stata’s survey package. The survey weights were calculated by dividing the total population by the sample size of each city. Crude and adjusted odds ratios (AORs) along with 95% confidence intervals (CI) were reported. Stata version 14.1 (College Station, Texas) was used for the analyses of these data. *P* values less than 0.05 were considered statistically significant.

## Results

### Participants’ characteristics

In both surveys, most participants were male (96.0% in 2010 and 98.7% in 2014), had an education less than high school (69.3% in 2010 and 67.0% in 2014), had a history of incarceration (81.2% in 2010 and 79.8% in 2014), and had low income levels (76.9% in 2010 and 39.9% in 2014). Among 1783 PWID in the 2010 survey, 790 (49.2%; 95% CI 46.3, 52.0) and, among 2166 PWID in the 2014 survey, 905 (45.8%; 95% CI 43.3, 48.4) reported past month OAT uptake. Overall, the prevalence of past month OAT uptake showed a significant decreasing trend (49.2% in 2010 vs. 45.8% in 2010, *P* = 0.033). The trend was decreasing among those with a history of incarceration (53.2% vs. 47.7%), those with no history of incarceration (48.2% vs. 38.8%), those who had ever been married (47.9% vs. 40.4%), and those who had low levels of self-perceived risk of HIV (56.1% vs. 44.3%). The past-month OAT uptake increased over time among PWID who only used non-opioid drugs in the previous month (2.0% vs. 32.9%) (Table 1).

**Table 1:** Characteristics and prevalence of past-month opioid agonist therapy (OAT) uptake among people who inject drug in Iran in two consecutive bio-behavioral surveillance surveys in 2010 (*N* = 1783) and 2014 (*N* = 2166)

		2010			2014			***P*** value*
Variables		***N*** (%)	People with OAT uptake ***N*** (%)	Past-month OAT uptake in 2010 %	***N*** (%)	People with OAT uptake ***N*** (%)	Past-month OAT uptake in 2014 %	
**Overall**		1783	790 (100.0)	49.2	2166	905 (100.0)	45.8	0.033
**Sex**	Male	1732 (96.0)	759 (94.9)	48.6	2120 (98.7)	881 (98.2)	45.6	0.063
	Female	51 (4.0)	31 (5.1)	63.2	46 (1.3)	24 (1.8)	64.4	0.902
**Age at interview **	≤35	1054 (57.3)	439 (54.1)	46.6	1075 (47.8)	403 (44.6)	42.8	0.077
	>35	725 (42.7)	351 (45.9)	52.9	1089 (52.2)	501 (55.4)	48.6	0.072
**Marital status **	Single (never married)	816 (44.9)	355 (43.8)	47.9	1000 (44.4)	380 (39.1)	40.4	0.001
	Ever Married	967 (55.1)	455 (56.2)	50.2	1105 (55.6)	501 (60.9)	50.3	0.963
**Income**	≤ 200 USD (6,000,000 Rials)	1396 (76.9)	630 (78.0)	49.9	834 (39.9)	332 (40.0)	45.9	0.067
	> 200USD (6,000,000 Rials)	331 (23.1)	138 (22.0)	47.0	1171 (60.1)	500 (60.0)	44.8	0.477
**Education level**	High school and above	544 (30.7)	244 (29.0)	46.4	696 (33.0)	283 (31.7)	44.0	0.399
	Less than high school	1237 (69.3)	545 (71.0)	50.4	1469 (67.0)	621 (68.3)	46.7	0.055
**History of incarceration (ever)**	Yes	1418 (81.2)	624 (79.6)	53.2	1651 (79.8)	716 (82.9)	47.7	0.002
	No	359 (18.8)	164 (20.4)	48.2	513 (20.2)	189 (17.1)	38.8	0.005
Substance use type in the past month**	Only opioids	1218 (77.1)	584 (87.9)	51.5	384 (25.8)	174 (29.3)	46.7	0.100
	Only non-opioids	126 (8.0)	3 (0.4)	2.0	119 (10.8)	32 (8.6)	32.9	<0.001
	Opioids and non-opioids	213 (14.9)	69 (11.7)	35.6	967 (63.4)	352 (62.1)	40.3	0.203
**Self-perceived risk of HIV**	Yes	960 (58.9)	393 (53.6)	45.2	954 (49.1)	431 (50.9)	47.5	0.286
	No	649 (41.1)	328 (46.4)	56.1	1204 (50.9)	469 (49.1)	44.3	<0.001
**HIV status**	Negative	1408 (85.0)	566 (81.1)	44.5	1911 (92.3)	768 (90.0)	44.9	0.818
	Positive	227 (15.0)	129 (18.9)	59.6	171 (7.7)	98 (10.0)	58.3	0.795
**Source of recruitment**	Outreach	142 (8.0)	43 (5.5)	38.8	363 (16.8)	184 (13.4)	48.2	0.056
	Facility-based	1641 (92.0)	747 (94.5)	50.0	1801 (83.2)	720 (86.6)	45.4	0.007

*P values are for comparison of prevalence between two rounds of surveys in 2010 and 2014

**Type of substance: non-opioids = Shishe, hashish/grass/cannabis, marijuana, ecstasy, cocaine, and methamphetamine/crystal; opioids = opium, opium sap, opium syrup, heroin, norchizak, tamchizak, buprenorphine, methadone, and crack

### Past-month OAT uptake in subgroups

**Fig. 1:**
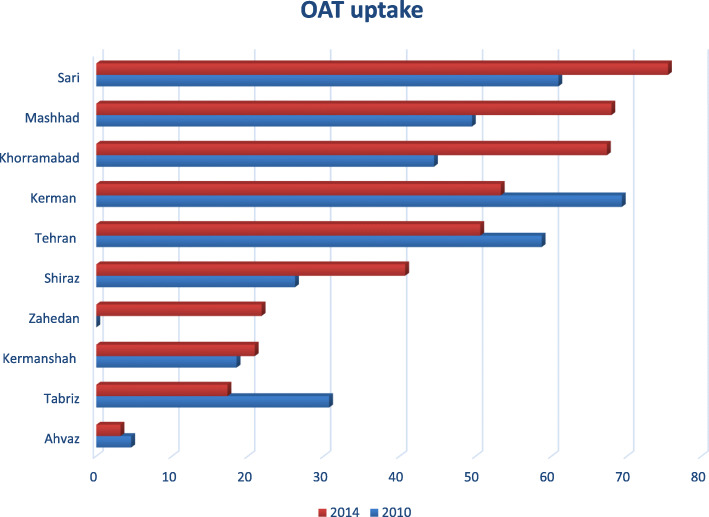
OAT uptake in different cities in Iran in 2010 and 2014 national bio-behavioral surveillance surveys

In both surveys, the prevalence of past-month OAT uptake was higher among people who had ever been married (50.2% in 2010 vs. 50.3% in 2014), were older than 35 years (52.9% in 2010 vs. 48.6% in 2014), were living with HIV (59.6% in 2010 vs. 58.3% in 2014), and had a history of incarceration (53.2% in 2010 vs. 47.7% in 2014). Conversely, the prevalence of OAT uptake was lower among those who reported last-month non-opioid drug use (2% in 2010 vs. 32.9% in 2014) (Table 1). Moreover, OAT prevalence varied across the studied cities, ranging from 0% in Zahedan to 69.4% in Kerman in 2010 survey and 3.2% in Ahvaz to 75.5% in Sari in 2014 survey (Fig. 1).

### OAT-associated factors

In the multivariable model, being ever married (AOR = 1.40; 95% CI 1.12, 1.75), HIV sero-positivity (AOR = 1.63; 95% CI 1.08, 2.50), and incarceration history (AOR = 1.56; 95% CI 1.16, 2.09) increased the odds of receiving OAT while last-month non-opioid drug use (AOR = 0.43; 95% CI 0.26, 0.71) and last-month concurrent opioid and non-opioid drugs use (AOR = 0.66; 95% CI 0.51, 0.86) decreased the odds of receiving OAT (Table 2).

**Table 2:** Correlates of currently receiving opioid agonist therapy (OAT) among people who inject drug in Iran in 2014

Variables		Crude OR (95% CI)	***P*** value	Adjusted OR (95% CI)	***P*** value
**Sex**	Male	1			
	Female	2.17 (1.07, 4.37)	0.031		
**Age at interview **	>35	1.26 (1.02, 1.55)	0.026		
	≤35	1			
**Marital status **	Single (never married)	1		1	
	Ever married	1.35 (1.13, 1.61)	0.001	1.40 (1.12, 1.75)	0.004
**Income**	≤200 USD (6,000,000 Rials)	1			
	>200 USD (6,000,000 Rials)	0.93 (0.75, 1.17)	0.569		
**Education level**	High school and above	1			
	Less than high school	1.11 (0.89, 1.38)	0.336		
**History of incarceration (ever)**	Yes	1.43 (1.12, 1.84)	0.005	1.56 (1.16, 2.09)	0.003
	No	1		1	
Substance use type in the past month**	Only opioids	1		1	
	Only non-opioids	0.55 (0.33, 0.92)	0.025	0.43 (0.26, 0.71)	0.001
	Opioids and non-opioids	0.76 (0.57, 0.92)	0.072	0.66 (0.51, 0.86)	0.001
**Self-perceived risk of HIV**	Yes	1.13 (0.92, 1.40)	0.223		
	No	1			
**HIV status**	Negative	1		1	
	Positive	1.72 (1.15, 2.56)	0.009	1.63 (1.08, 2.50)	0.022
**Source of recruitment**	Outreach	1			
	Facility-based	0.89 (0.67, 1.19)	0.458		

**Type of substance: non-opioids = Shishe, hashish/grass/cannabis, marijuana, ecstasy, cocaine, and methamphetamine/crystal; opioids = opium, opium sap, opium syrup, heroin, norchizak, tamchizak, buprenorphine, methadone, and crack

## Discussion

Our findings showed that as of 2014, less than half of PWID in Iran received OAT in the previous year with significant heterogeneity in OAT uptake across cities. Being ever married, HIV positive, and having a history of incarceration were positively associated with receiving OAT, while using non-opioid drugs were negatively associated with receiving OAT.

Moreover, we demonstrated that less than half of the surveyed PWID used OAT in the previous year. Based on the World Health Organization's (WHO) definition of high coverage of OAT (i.e., 40% or more), Iran falls into the high coverage category [16]. However, there is a high level of disparity for OAT uptake across cities with OAT uptake ranging from 0-75% in different cities. Interestingly, all cities with low OAT coverage were among the less and under developed regions, settings that also have higher rates of child mortality and lower numbers of rehabilitation centers and paramedics in comparison with the rest of the country [17]. Therefore, to reach and maintain the high coverage goal in all regions of the country, allocation of resources regarding the degree of inequality in the distribution of OAT services should be considered in future planning and financing of these services. In addition, addressing and removing the potential barriers to access and use of OAT such as financial barriers, lack of awareness and negative attitudes, worries about methadone’s side effects, and social stigma attached to receiving OAT are integral to increasing the coverage rate of OAT uptake among Iranian PWID [8]. Tackling barriers to OAT access are of particular importance in the context of COVID-19 and future pandemics as accessing such services among PWID is often accentuated during health emergencies. Comparing our results to other countries of the MENA region, the OAT uptake in Iran seems to be higher than most of its neighboring countries. Indeed, the overall OAT provision in MENA is very limited. For example, In 2017, only 7 MENA countries provided OAT which suggests ~ 6% of PWID in the MENA region to be on OAT [18]. However, due to non-random nature of our study sample, these comparisons should be interpreted with caution.

The OAT uptake in PWID slightly decreased in 2014 in comparison with 2010. This trend is in opposite direction with the increasing number of facilities (from 700 centers in 2007 to 3373 centers in 2014) [19] that provide OAT services to PWID. Although the observed pattern may be simply due to possible biases in selection of the participants, it might also be due to the recent shift in substance use practices among PWID in Iran and the increase in poly-drug use involving non-opioids among them. Recent studies have shown that methamphetamine use has been increasing among PWID with opioid use disorder [20]. We also found that compared to PWID who used only opioids within the previous month, those who used only non-opioids and those who used opioids and non-opioids simultaneously, were less likely to have received OAT. Therefore, one possible explanation for the decreasing trend of OAT could be PWID's increasing tendency toward poly-drug use including stimulants.

Following the emergence and increasing supply of synthetic non-opioid drugs including methamphetamines, more PWID tend to use these drugs [21]. On the other hand, the use of methamphetamine in PWID reduces the effectiveness of OAT programs and subsequently leads to lower satisfaction of patients with OAT [22]. These issues are problematic in a way that treatment of PWID who use synthetic drugs has turned into a challenging issue within the last few years [20, 21]. In Iran, there are only a limited number of centers providing treatments for stimulant use disorder. Preliminary studies indicate that the integration of stimulant HR services into opioid HR programs at DICs could be an effective strategy in reducing high-risk behaviors of their clients [23]. Therefore, policies toward the establishment of such centers and providing treatments for stimulant use disorder at DICs should be considered in future policy and planning across the country.

Living with HIV was associated with an increased likelihood of OAT uptake, a finding which is consistent with a study conducted in Vancouver, Canada [24]. This may be partly due to the effect of post-test counseling which is freely available for all PWID who undergo HIV testing in Iran. Integration of HIV and substance use services have been shown to improve HIV treatment and care continuum among PWID living with HIV [25, 26].

In our study, having a history of incarceration was positively associated with OAT uptake. This may be due to the establishment of HR programs inside Iran’s prisons. Similar to several international settings, people who use drugs are overrepresented in prisons across Iran [27, 28]. More than 50% of all Iranian prisoners are being held on drug-related offenses and 70% of them use illicit drugs [29]. When Iran experienced large outbreaks of HIV among incarcerated populations in the early 2000s, HR programs inside prisons were rapidly expanded. As these HR provision and coverage continue to function inside prisons, most PWID with a history of incarceration are likely to have used these services and received OAT during their incarceration period. Previous studies have shown that exposure of prisoners to OAT inside prisons increases their chance of receiving OAT even after their release [18, 30]. Therefore, ensuring the continuation and extension of current strategies of HR inside prisons in Iran is of utmost importance.

We acknowledge the limitations of our study. First, social desirability bias may have resulted in over-reporting of OAT uptake and under-reporting of stigmatized and criminalized behaviors such as use of drugs and alcohol. Second, the study was cross-sectional with limited capacity for causal inference. Third, male PWID were overrepresented in our study sample and our findings may not be generalizable to female PWID in Iran. Fourth, due to non-random selection of the study participants and the possible role of selection bias, the findings might not necessarily represent OAT uptake among all PWID in Iran. Fifth, our data was collected in late 2014 and was delayed in getting published due to several contextual and logistical complexities; therefore, it might not provide a realistic picture of the current status of OAT uptake among PWID in Iran. Lastly, differences in sampling strategy between two study rounds including recruiting participants from different facilities and sites cannot be ruled out as unmeasured confounders, and therefore, comparison between the two rounds should be made with caution.

## Conclusion

Despite the high level of OAT uptake among PWID, the level of heterogeneity in access to OAT in Iran is alarming. These data highlight the need to strengthen HR policies focused on providing equal access to OAT among all PWID. Furthermore, the OAT services appear to be serving PWID with a higher risk profile (e.g., those living with HIV and those with a history of incarceration). Therefore, service integration including mental health, HIV and HCV care, and other HR services may support optimal implementation and health-related impacts of OAT across Iran.

## Data Availability

The datasets used and/or analyzed during the current study are available from the corresponding author on reasonable request.

## Abbreviations

OAT: Opioid agonist therapy
PWID: People who inject drugs
HIV: Human immunodeficiency virus
HCV: Hepatitis C virus
VCT: Voluntary counseling and testing
BMT: Buprenorphine maintenance therapy
MENA: Middle East and North Africa
MMT: Methadone maintenance treatment
STI: Sexually transmitted infections
HBV: Hepatitis B virus
NSPs: Needle and syringe programs
HR: Harm reduction
BBSS: Bio-behavioral surveillance surveys
WHO: World Health Organization
DICs: Drop-in centers
